# Comparative Biophysical and Ultrastructural Analysis of Melanins Produced by Clinical Strains of Different Species From the Trichosporonaceae Family

**DOI:** 10.3389/fmicb.2022.876611

**Published:** 2022-04-25

**Authors:** Iara Bastos de Andrade, Glauber Ribeiro de Sousa Araújo, Fábio Brito-Santos, Maria Helena Galdino Figueiredo-Carvalho, Rosely Maria Zancopé-Oliveira, Susana Frases, Rodrigo Almeida-Paes

**Affiliations:** ^1^Laboratório de Micologia, Instituto Nacional de Infectologia Evandro Chagas, Fundação Oswaldo Cruz, Rio de Janeiro, Brazil; ^2^Laboratório de Biofísica de Fungos, Instituto de Biofísica Carlos Chagas Filho, Universidade Federal do Rio de Janeiro, Rio de Janeiro, Brazil; ^3^Rede Micologia RJ – FAPERJ, Rio de Janeiro, Brazil

**Keywords:** Trichosporonaceae, melanin, virulence, biophysics, *Trichosporon asahii*

## Abstract

Melanin is one of the most studied virulence factors in pathogenic fungi. This pigment protects them from a series of both environmental and host stressors. Among basidiomycetes, *Cryptococcus neoformans* and *Trichosporon asahii* are known to produce melanin in the presence of phenolic precursors. Other species from the Trichosporonaceae family also produce this pigment, but the extent to this production among the clinically relevant species is unknown. For this reason, the aim of this study was to verify the production of melanin by different Trichosporonaceae species of clinical interest and to compare their pigments with the ones from *C. neoformans* and *T. asahii*, which are more prevalent in human infections. Melanin was produced in a minimal medium supplemented with 1 mM L-dihydroxyphenylalanine (L-DOPA). Pigment was evaluated using scanning electron microscopy, Zeta potential measurements, and energy-dispersive X-ray spectroscopy. It was found that, besides *C. neoformans* and *T. asahii*, *Trichosporon japonicum*, *Apiotrichum montevideense*, *Trichosporon inkin*, *Trichosporon faecale*, *Cutaneotrichosporon debeurmannianum*, and *Cutaneotrichosporon arboriformis* also produce melanin-like particles in the presence of L-DOPA. Melanin particles have negative charge and are smaller than original cells. Variations in color, fluorescence, and chemical composition was noticed between the studied strains. All melanins presented carbon, oxygen, sodium, and potassium in their composition. Melanins from the most pathogenic species also presented iron, zinc, and copper, which are important during parasitism. Biophysical properties of these melanins can confer to the Trichosporonaceae adaptive advantages to both parasitic and environmental conditions of fungal growth.

## Introduction

The Trichosporonaceae family comprises a large number of yeast-like basidiomycetes that are widely distributed in nature. Some of them are part of the skin microbiota and gastrointestinal tract of healthy humans (Colombo et al., 2011). Fungi of this family can cause a wide-range of clinical manifestations, from the superficial mycosis white piedra to systemic infections, usually in immunocompromised patients (Duarte-Oliveira et al., 2017). For a long time, the genus *Trichosporon* was accounted for these infections. However, with the advent of the molecular biology, a taxonomic reclassification of this genus resulted in the distribution of some *Trichosporon* species to other fungal genera, such as *Apiotrichum* and *Cutaneotrichosporon*. The species in this family with a major clinical relevance include *Trichosporon asahii, Trichosporon inkin, Trichosporon faecale*, and *Trichosporon asteroides* (Liu et al., 2015; Wang and Wang, 2015).

The ability of pathogenic species of the Trichosporonaceae family to cause invasive infections is associated to some virulence factors, which allow the fungus to change from commensalism to a parasitic stage, causing disease when a disturbance in the host homeostasis occurs (Mariné et al., 2015). The main virulence factors of these species are the production of extracellular enzymes, biofilm formation, resistance to antifungal drugs and oxidizing agents, metabolic plasticity, phenotypic switching, and the presence of certain molecules such as glucuronoxylomannan and melanin on the fungal cell wall (Duarte-Oliveira et al., 2017).

Among these virulence factors, melanin is extremely important because this pigment protects several fungi from the host immune system and antifungal drugs. In addition, melanin plays an important role as a protector from environmental adverse conditions (Taborda et al., 2008). It has been found that melanins give to fungi the ability to tolerate extreme environments such as the Earth’s poles and places contaminated by heavy metals, ionizing radiation, and toxic substances (Cordero and Casadevall, 2017). A notable property of melanin is its ability to interact with a wide range of electromagnetic radiation frequencies, acting as a shield and converting radiation into energy to growth (Casadevall et al., 2017). The most studied fungus regarding melanization is *Cryptococcus neoformans*, a basidiomycetous yeast that causes a life-threatening fungal meningitis (Casadevall and Smith, 2019). A study about melanization in the Trichosporonaceae family revealed that *Trichosporon mucoides*, *T. asahii*, *T. inkin*, and *T. asteroides* are able to produce melanin using L-dihydroxyphenylalanine (L-DOPA) as a precursor (Figueiredo-Carvalho et al., 2014). However, the extent of this knowledge to other species of clinical interest from this family is unknown.

Three types of melanins have been described in human pathogenic fungi, which are eumelanin, formed from DOPA-quinone, pyomelanin, derived from homogentisic acid yielded by tyrosine catabolism, and allomelanins, formed from nitrogen free precursors. In fungi, dihydroxynaphtalene (DHN)-melanin is the major allomelanin type, and it is derived from 1,8-DHN generated by a series of reactions starting with the polymerization of endogenous acetyl-CoA or malonyl-CoA into 1,3,6,8-tetrahydroxynaphthalene by a polyketide synthase (Cao et al., 2021). Melanins have distinct biophysical properties that make them interesting and unique biomolecules. They are hydrophobic, negatively charged macromolecules with high molecular weight (Frases et al., 2007). Melanins usually absorb light in the UV spectrum. In fact, studies to characterize melanin apply the UV-visible absorption spectrum for most types of this pigment. The maximum wavelength of absorption of alkaline solutions varies between 196 and 300 nm, depending on the source of melanin (Pralea et al., 2019). Melanin’s peak of ultraviolet light absorption is apparently due to the complex conjugate molecules that confer to this structure the ability to absorb and scatter ultraviolet light photons (Hou et al., 2019).

Although dozens of *Trichosporon* species have been reported as agents of human infections, *T. asahii*stands as the major agent of trichosporonosis worldwide (Francisco et al., 2019). The reasons for the high prevalence of this species are not well established. Biophysical properties of cell surface and their components, such as melanin, are associated with virulence in fungal pathogens (Rosas Ál Nosanchuk et al., 2000; Gómez and Nosanchuk, 2003). One hypothesis is that Trichosporonaceae species have melanin as a virulence factor that could facilitate its parasitism Therefore, the aim of this study was to compare, by ultrastructure and biophysical methods, melanins produced by six different species of clinical interest of the *Trichosporonaceae* family with the melanin produced by *T. asahii*, as well as with the melanin produced by *C. neoformans*, a highly pathogenic basidiomycetes that also produces melanin from L-DOPA.

## Materials and Methods

### Fungal Strains

Ten different clinical isolates from the Trichosporonaceae family, all from the Coleção de Fungos Patogênicos, Fiocruz (WDCM 951) were analyzed in this study. They include *Trichosporon asahii* (CFP00944), *Trichosporon japonicum* (CFP00907), *Trichosporon inkin* (CFP00904, CFP00946, and CFP00951), *Trichosporon faecale* (CFP00905), *Apiotrichum montevideense* (CFP00909 and CFP00950), *Cutaneotrichosporon debeurmannianum* (CFP00913), and *Cutaneotrichosporon arboriformis* (CFP00914). All strains were identified by molecular methods, as previously described (Andrade, 2019). The *Cryptococcus neoformans* var. grubii H99 strain (ATCC 208821) was included in the analyses as a positive melanization control.

### *In vitro* Melanization Assay

The strains were cultured on Potato Dextrose Agar for 48 h at 25°C. Fungal cells were used to inoculate a chemically defined minimal medium for melanin production (15 mM glucose, 10 mM MgSO_4_, 29.4 mM KH_2_PO_4_, 13 mM glycine, 3 μM thiamine, 1 mM L-DOPA, pH 5.5), in a final concentration of 5 × 10^4^ cells/mL, as previously described (Figueiredo-Carvalho et al., 2014). Control cultures consisted of the same number of cells inoculated on minimal medium without L-DOPA supplementation.

### Melanin Extraction

The cultures mentioned above were incubated in the dark for 14 days at 37°C under agitation (150 rpm). Fungal cells were collected by centrifugation at 10,842 × *g* for 13 min, washed three times with phosphate-buffered saline (PBS) (pH 7.0), and washed in 1.0 M sorbitol, 0.1 M sodium citrate (pH 5.5). Cells were harvested, lytic enzymes from *Trichoderma harzianum* (Sigma-Aldrich, St. Louis, MO, United States) were added at 10 mg/ml, and the suspension was incubated at 30°C for 1 h to generate fungal protoplasts. The protoplasts were collected by centrifugation, washed three times with PBS, and incubated with 4.0 M guanidine thiocyanate (Sigma-Aldrich), a denaturant agent, for 1 h, with frequent vortexing. Cell debris were harvested, washed three times with PBS, and then boiled in 6.0 M HCl (Sigma-Aldrich) for 1 h. Melanin particles were collected by centrifugation, washed extensively with PBS, and stored at 4°C. As per comparison purposes, 500 μl of each melanin suspension, including controls, were added to microcentrifuge tubes for a pigment intensity visual analysis, as described for other fungi (Rosas Ál Nosanchuk et al., 2000).

### Melanin Quantification

The extracted melanins were quantified in 96-well microplates, in a final volume of 100 μL per well. For this, the optical density (OD) of the wells at 340 nm was determined using the Epoch microplate spectrophotometer (BioTek, Winooski, VT, United States), both for the melanin particles and for the negative control, medium with L-DOPA without cells. This experiment was performed in triplicate.

### Scanning Electron Microscopy

The melanin particles were adhered to 12 mm diameter round glass coverslips (#1, borosilicate glass of hydrolytic class 1 – Knittel Glasbearbeitungs GmbH, Germany) previously coated with 0.01% poly-L-lysine (Sigma-Aldrich, Darmstadt, Germany) for 60 min and dried in a nitrogen-rich atmosphere. Finally, they were mounted on stubs and coated with a thin layer gold-palladium (10–15 nm) using the sputtering method (Balzers Union FL-9496, Balzers, FL) and viewed on a scanning electron microscope (FEI Quanta FEG 450) at a voltage of 10 kV.

### Melanin Composition

A scanning electron microscopy with energy-dispersive X-ray spectroscopy (EDS) was performed to determine the fundamental composition of the melanin constituents of the samples. This consists of identifying the lines in the X-ray spectrum using energy tables or wavelengths and quantitative analysis of EDS (determination of the concentrations of the elements present) by measuring (run time was approximately 45 s) the line intensities for each element of the sample and for the same elements in the known composition calibration standards. As a control a carbon adhesive (Ted Pella Spectro Tabs, High Purity Conductive Carbon Tabs, 12 mm O.D., #16084-4) mounting stub was analyzed without particles, and the resulting spectra showed no contributions from the stub, i.e., aluminum (Al) or sulfur (S).

Microscopy was performed on the FEI Quanta FEG 450 with Apollo X – EDAX scanning microscope. Analyses were performed at a 10,000× magnification using the SEM’s backscattered electron (BSE) detector.

### Zeta Potential (ζ) and Conductance

These measurements were conducted in a Zeta potential analyzer NanoBrook Omni (Brookhaven Instruments Corp., Holtsville, NY, United States) as previously described (Almeida-Paes et al., 2012). Ten measurements for each sample were made at 25°C.

### UV-Visible Light Absorption Spectrum

Melanins were suspended in PBS after vigorous vortexing as the reference. These samples were distributed (100 μl) in a 96-well UV transparent microplate. The UV – visible absorption spectrum of the melanin was scanned in the wavelength range of 200–700 nm with the UV – visible spectrophotometer SpectraMax plus 384 (Molecular Devices, San José, CA, United States). Absorbance values were used to construct the UV-light absorption spectrum of each melanin. Additionally, the logarithm of absorbance values were plotted against the 250–500 nm wavelengths and the slopes compared among the samples, as described (Pralea et al., 2019). This experiment was performed in triplicate.

### Fluorescence Microscopy

The particles recovered from brown cells after treatment with enzymes, denaturant, and hot acid were visualized under a 1,000× magnification using a fluorescence microscopy (Axio Observer, ZEISS, Germany) with DAPI, Alexa Fluor 488, Alexa Fluor 546, and Far-Red filters.

### Statistical Analyses

The software GraphPad Prism 7 (GraphPad Software, Inc., San Diego, CA, United States) was used for the analyses. Comparisons were made using the ANOVA test with Bonferroni’s multiple comparison test. Slopes of logarithmic spectral curves were compared after a linear regression analysis. A *p* < 0.05 was considered significant.

## Results

### Different Species of the Trichosporonaceae Family Produce Melanin

**FIGURE 1:**
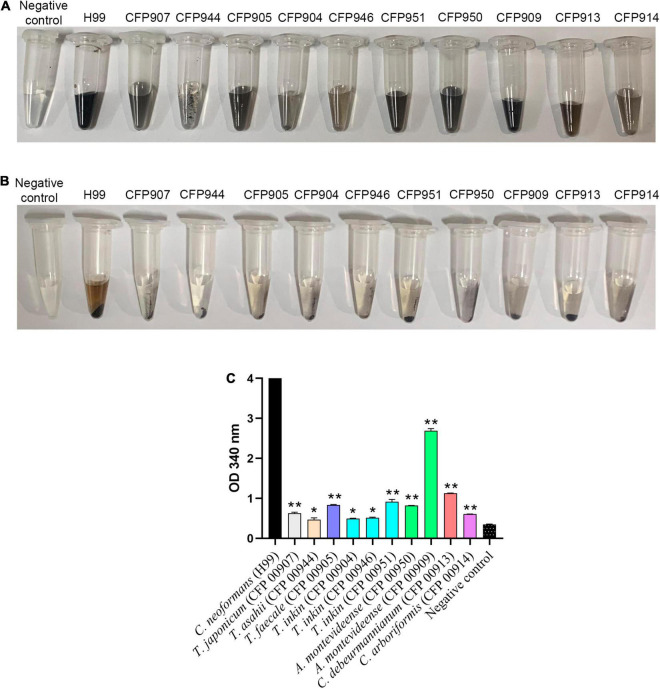
Production of melanin by 10 different Trichosporonaceae strains in the presence of L-DOPA. Each tube contains melanin ghosts of each strain cultured for 14 days with 1 mM L-DOPA. The H99 *Cryptococcus neoformans* strain was used as a positive control and minimal medium with 1 mM D-DOPA without cells as a negative control. **(A)** Vortex homogenized melanin particles. **(B)** Pellet of melanin particles. **(C)** Quantification of melanin by spectrophotometry. *Indicates a statistical difference when compared only with the *Cryptococcus neoformans* H99 melanin control. **Indicates a statistical difference when compared with the *Trichosporon asahii* and *Cryptococcus neoformans* grubii H99 melanin.

All studied species included in this work were able to produce melanin-like particles only when grown in the presence of L-DOPA. It was observed that production of pigment was variable among strains, as depicted in Figures 1A,B. Overall, it was visually seen that *C. neoformans* produced black melanin. Among the studied Trichosporonaceae strains, it was noticed that, like *C. neoformans* H99, one *A. montevideense* strain (CFP00909) produced black pigment. Four strains produced dark-brown pigment: *A. montevideense* (CFP00950), *C. debeurmannianum* (CFP00905), *T. inkin* (CFP00951), and *T. japonicum* (CFP00907). The other *C. debeurmannianum* strain (CFP00913) produced brown pigment. Three strains produced light-brown pigment: *T. inkin* (CFP00904 and CFP00946) and *C. arboriformis* (CFP00914). The *T. asahii* strain (CFP00944) produced black melanin particles (Figure 1B), but in lower amounts than *C. neoformans* and *A. montevideense*. Overall, it was observed that *C. neoformans* produced more melanin than all members of the Trichosporonaceae family (*p* < 0.05). When compared to *T. asahii*, only the samples of *Trichosporon inkin* (CFP00904 and CFP00946) had no significant difference (Figure 1C).

### Ultrastructure of Melanin Particles Surface

**FIGURE 2:**
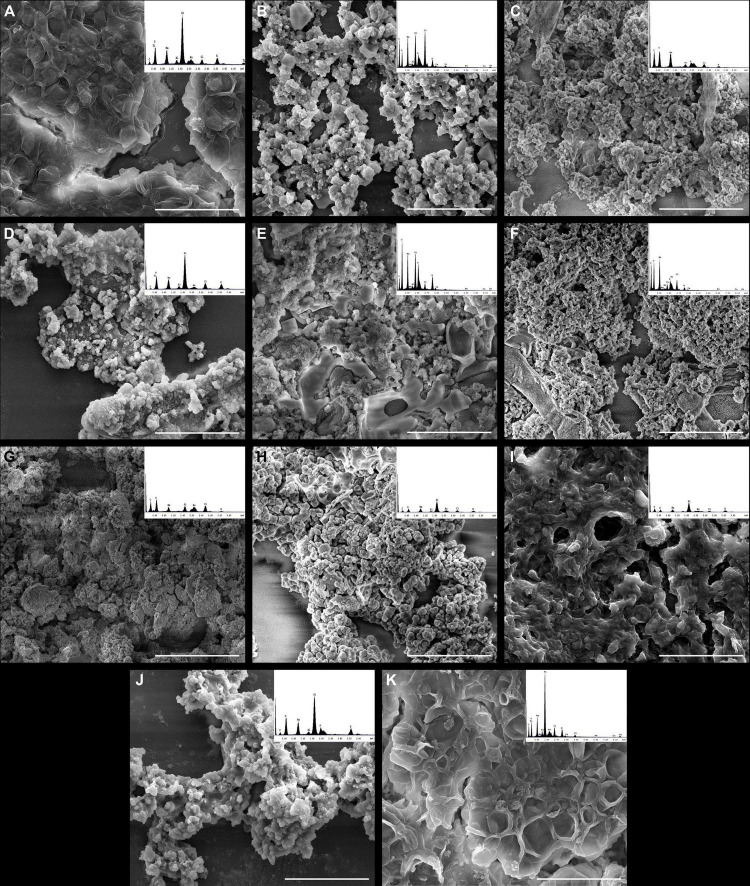
High-resolution scanning electron microscopy and energy-dispersive X-ray spectroscopy (EDS) of melanin produced by clinical strains of different species of the Trichosporonaceae family. **(A)** *Trichosporon asahii* (CFP00944), **(B)** *Trichosporon japonicum* (CFP00907), **(C)** *Trichosporon faecale* (CFP00905), **(D)** *Trichosporon inkin* (CFP00904), **(E)** *Trichosporon inkin* (ATCC 18020) (CFP00946), **(F)** *Trichosporon inkin* (CFP00951), **(G)** *Apiotrichum montevideense* (CFP00950), **(H)** *Apiotrichum montevideense* (CFP00909), **(I)** *Cutaneotrichosporon debeurmannianum* (CFP00913), **(J)** *Cutaneotrichosporon arboriformis* (CFP00914), and **(K)** *Cryptococcus neoformans* var. grubii H99. The EDS histograms are presented at the upper right corner of each panel and in a larger scale at the Supplementary Figure 1. Scale bar = 10 μm.

The melanin particles of all Trichosporonaceae strains studied were grouped into lumps with different shapes and dimensions (Figure 2) differing from original cells, which are ovoid to globose, measuring around 3 × 5 μm (Kwon-chung and Bennett, 1992).

### Composition of Melanins From Different Species of the Trichosporonaceae Family

Supplementary Figure 1 presents the energy dispersive spectroscopy (EDS) of melanins produced by clinical strains of different species from the Trichosporonaceae family, which permitted the determination of the elemental composition of melanins. The composition of melanins from the different studied species is presented in Table 1. Sixteen chemical elements were found among the different strains. However, the number of different elements differed among them. For instance, melanins from *C. debeurmannianum* (CFP00913), *A. montevideense* (CFP00950), and *T. faecale* (CFP00905) presented six elements, while melanins from *T. asahii* (CFP00944) and *T. inkin* (CFP00951) presented 13 different elements. Overall, all samples presented high carbon, oxygen, and sodium concentrations. Potassium was also found, in lower proportion, in all samples. Nitrogen and sulfur were not found in any studied melanin. A few samples presented the heavy metals barium, vanadium, and titanium. Iron, copper, and zinc were observed in *T. asahii* and *T. inkin* melanins.

**TABLE 1:** Composition of chemical elements present in Trichosporonaceae and *C. neoformans* melanin samples.

	Elements																	
Sample	C	O	Na	K	Mg	Al	P	Au	Cl	Ca	Ti	Ba	V	Fe	Cu	Zn	Total%
*T. japonicum* (CFP00907)	17.19	38.75	14.55	6.34	0	2.66	0	13.77	4.12	0	2.43	0	0.19	0	0	0	100
*T. asahii* (CFP00944)	51.78	17.70	10.75	2.42	0.44	1.02	3.40	0	9.91	0.22	0.73	0	0	0.09	0.08	1.45	100
*T. faecale* (CFP00905)	44.16	35.54	14.23	0.94	0	0	2.99	0	2.14	0	0	0	0	0	0	0	100
*T. inkin* (CFP00904)	10.41	42.68	15.79	7.61	0	2.95	0	9.79	8.29	0	2.48	0	0	0	0	0	100
*T. inkin* (CFP00946)	24.98	42.48	15.83	4.97	0	0.82	7.96	0	1.92	0.13	0	0	0	0.10	0.12	0.69	100
*T. inkin* (CFP00951)	40.93	29.37	13.94	1.08	0.54	0.54	2.86	6.58	3.32	0.14	0	0	0	0.20	0.25	0.24	100
*A. montevideense* (CFP00950)	40.13	34.43	6.18	0.73	0	0	0	12.93	5.60	0	0	0	0	0	0	0	100
*A. montevideense* (CFP00909)	27.78	27.83	13.12	5.03	0	2.25	0	18.09	5.88	0	0	0	0	0	0	0	100
*C. debeurmannianum* (CFP00913)	48.50	23.37	9.08	5.34	0	0	0	10.88	2.82	0	0	0	0	0	0	0	100
*C. arboriformis* (CFP00914)	18.19	43.58	16.37	6.70	0	2.19	7.27	0	0	0	0	5.70	0	0	0	0	100
*C. neoformans* (H99)	38.21	24.16	12.06	4.47	0.11	2.08	0	9.09	4.69	0.05	1.79	0	0	0.11	0	3.19	100

*Composition analysis performed by Scanning Electron Microscopy with X-ray spectroscopy by energy dispersion, in the FEI Quanta FEG 450 with Apollo X – EDAX scanning microscope (Mag. 10,000×).*

### Biophysical Properties of the Trichosporonaceae Melanin

**FIGURE 3:**
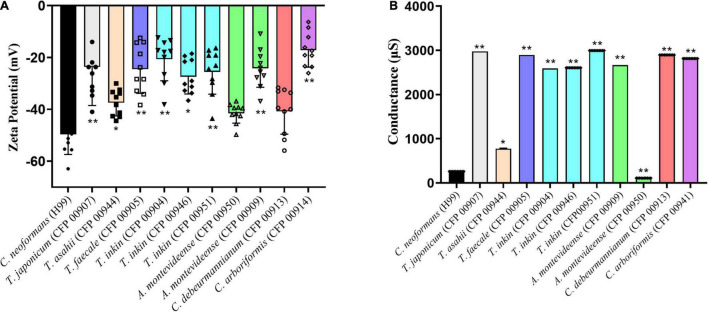
Biophysical properties of Trichosporonaceae melanin. **(A)** Zeta potential and **(B)** conductance of melanin particles obtained from cells grown with L-DOPA supplementation. Ten independent analyses were made for each strain. * Indicates a statistical difference when compared only with the *Cryptococcus neoformans* var. grubii H99 melanin control. ** Indicates a statistical difference when compared with the *Trichosporon asahii* and *Cryptococcus neoformans* var. grubii H99 melanin.

**FIGURE 4:**
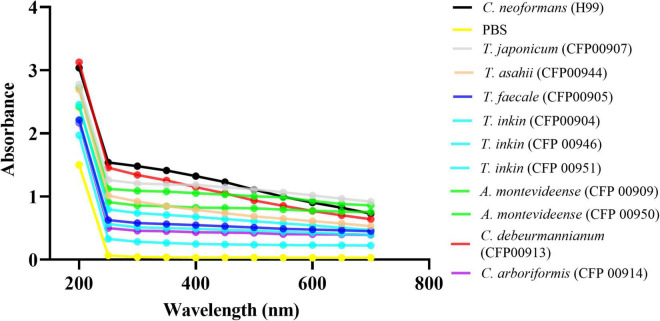
The UV-visible spectra of melanins from 10 different Trichosporonaceae strains. The *Cryptococcus neoformans* var. grubii H99 strain was used as a control. The optical densities to construct the spectra were obtained in triplicate.

**FIGURE 5:**
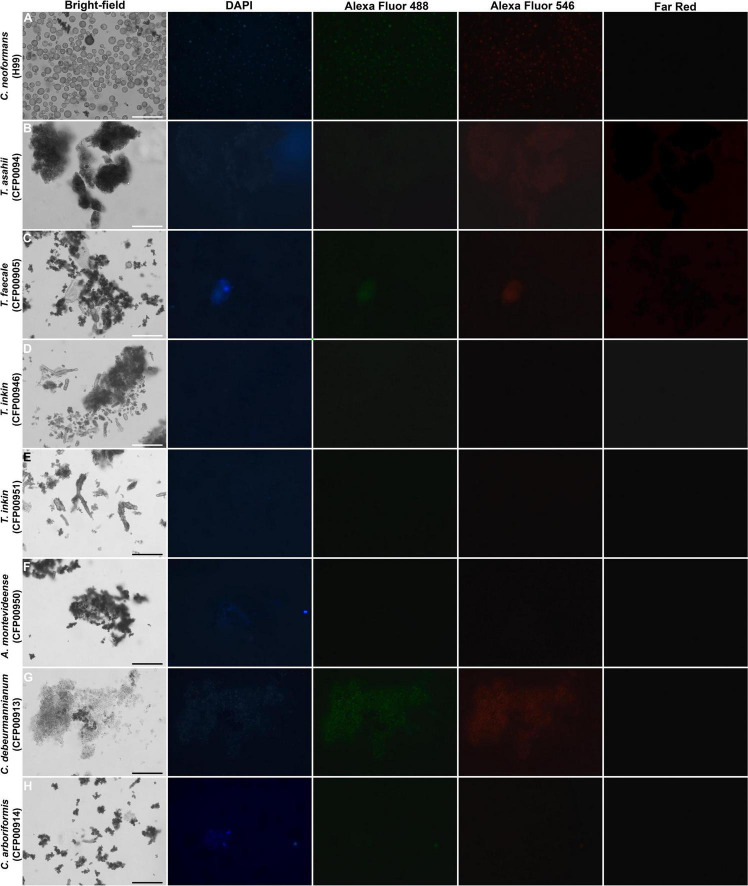
Fluorescence of particles recovered after HCl treatment from Trichosporonaceae cells grown in minimal medium supplemented with 1 mM of L-DOPA, shown by bright-field, fluorescence under a DAPI (4′,6′-diamidino-2-phenylindole), Alexa Fluor 488, Alexa Fluor 546, and Far-Red filters. Bar = 30 μm. **(A)** *Cryptococcus neoformans* H99, **(B)** *Trichosporon asahii* CFP00944, **(C)** *Trichosporon faecale* CFP00905, **(D)** *Trichosporon inkin* CFP00946, **(E)** *Trichosporon inkin* CFP00951, **(F)** *Apiotrichum montevideense* CFP00950, **(G)** *Cutaneotrichosporon debeurmannianum* CFP00913, **(H)** *Cutaneotrichosporon arboriformis* CFP00914.

The Zeta potential of melanin produced by *C. neoformans* was lower than most of members of the Trichosporonaceae family (*p* < 0.05), with the exception of the strains *C. debeurmannianum* (CFP00913) and *A. montevideense* (CFP00950). In addition, the zeta potential of melanin produced by *T. asahii* was lower than other Trichosporonaceae siblings, except *C. debeurmannianum* (CFP00913), *T. inkin* (CFP00946) and *A. montevideense* (CFP00950) (Figure 3A). The conductance was significantly different in the species of the family Trichosporonaceae when compared to *C. neoformans* (Figure 3B). Most Trichosporonaceae present high conductance (>2000 μS), except for *T. asahii* (766.1 ± 3.6 μS) and *A. montevideense* (108.4 ± 0.9 μS). All melanin samples exhibited UV spectra with absorption peaks around 200 nm followed by a progressive decrease for higher wavelengths (Figure 4). Table 2 presents the spectral characteristics for melanins produced by different strains. In addition, melanin particles were evaluated for their fluorescence properties under different wavelengths. Fluorescence was observed for seven strains when the particles were irradiated with light of 405, 495, 556, and 633 nm (Figure 5).

**TABLE 2:** Characteristics of the UV-Vis spectrum of melanins from 10 different strains of Trichosporonaceae.

Melanin strains	Maximum absorption wavelength (nm)	Slope
*C. neoformans* (H99)	200	−0.0005
*T. japonicum* (CFP00907)	200	−0.0002
*T. asahii* (CFP00944)	200	−0.0007
*T. faecale* (CFP00905)	200	−0.0003
*T. inkin* (CFP00904)	200	−0.0004
*T. inkin* (CFP00946)	200	−0.0004
*T. inkin* (CFP00951)	200	−0.0002
*A. montevideense* (CFP00950)	200	−0.0001
*A. montevideense* (CFP00909)	200	−0.0001
*C. debeurmannianum* (CFP00913)	200	−0.0007
*C. arboriformis* (CFP00914)	200	−0.0002

*The Cryptococcus neoformans var. grubii H99 strain was used as a control.*

## Discussion

Studies on the melanization of the *Trichosporon* genus are scarce, especially when considering the new taxonomic criteria of the Trichosporonaceae and its new genera. Our group described previously the production of eumelanin in *T. asahii*, *T. asteroides*, *T. inkin*, and *T. mucoides* (Figueiredo-Carvalho et al., 2014). The present study corroborated that *T. asahii* and *T. inkin* produce melanin in the presence of L-DOPA (Supplementary Figure 2) using new biophysical methods and added *T. japonicum*, *T. faecale*, *A. montevideense*, *C. debeurmannianum*, and *C. arboriformis* to the list of eumelanin producers from the Trichosporonaceae family.

Our results showed that all species included in this study were able to produce melanin-like pigment when the culture medium was supplemented with L-DOPA. Without L-DOPA all strains were completely digested by the denaturant, hot-acid treatment, indicating that this pigment is not DHN-melanin, the most common type of melanin in human pathogenic fungi, but eumelanin, similarly to *C. neoformans*. The pigment color ranged from brown to black depending on the study sample. Our results do not permit to conclude about quantities of melanin produced by different Trichosporonaceae species, but suggest that variations among and within species may occur. For instance, one *T. inkin* strain produced dark-brown melanin, while the other two produced light-brown melanin. Similar behavior of variation in melanin production within a single species was observed previously for other human pathogenic fungi like *Sporothrix brasiliensis* (Almeida-Paes et al., 2015) and the *C. neoformans/gattii* species complex (Oliveira et al., 2021).

Unlike *C. neoformans* melanin particles, which retain cellular shape and size after hot-acid treatment (Wang et al., 1996), melanin from Trichosporonaceae species are smaller than original cells and tend to clump. Similar behavior was observed for other fungi, such as *Candida albicans* (Morris-Jones et al., 2005) and *Candida glabrata* (Almeida-Paes et al., 2021). These particles have shape and sizes that resemble fungal melanosomes, such as those observed in *Fonsecaea pedrosoi* (Alviano et al., 1991).

Other important biophysical aspect of all Trichosporonaceae melanin particles was their negative charge, as detected by zeta potential measurements. This is a typical characteristic for fungal melanins (Nosanchuk and Casadevall, 1997; Frases et al., 2007; Almeida-Paes et al., 2012). The *C. neoformans* control strain had a lower charge when compared to the Trichosporonaceae. *C. neoformans* is more frequent than members of the Trichosporonaceae family as an agent of human infections. Unlike the Trichosporonaceae, *C. neoformans* can cause invasive infection in immunocompetent individuals (Corrêa Pinheiro et al., 2019). One of the factors that might contribute to this is the higher electronegativity conferred by the *C. neoformans* melanin. Besides *T. asahii*, *C. debeurmannianum* (CFP00913) and *A. montevideense* (CFP00950) strains presented the most electronegative zeta potential among the Trichosporonaceae in this study. It has been described that *Cryptococcus* melanin is negative, which confers resistance against phagocytosis (Nosanchuk and Casadevall, 1997). *T. asahii* appears to be the most virulent species in the Trichosporonaceae family, as it is frequently found as the causative agent of trichosporonosis (Padovan et al., 2019). The species *C. debeurmannianum* and *A. montevideense* are not often described as important agents of trichosporonosis (Nath et al., 2018). Our results suggest that the electronegativity of melanin may confer some pathogenic potential to this species, but the absence of other virulence factors may impair this species to play a more important role in human infections.

The results of the present study show differences in melanin composition and/or structure among the strains studied. These differences could be observed through fluorescence and EDS scanning electron microscopy. Melanin is a moderately low quantum yield fluorophore when excited with UV or visible light, and this fluorescence can be weakened by its own broad spectral absorption (Gallas and Eisner, 1987). In addition, fluorescence in eumelanin is related to chemically distinct oligomeric units that can be selectively excited (Perna et al., 2009). Since differences in the number of fluorescent samples were observed, as well as in the excitation wavelengths of fluorescent melanins, we could presume that they have different oligomers in their compositions.

Studies about *Sepia officinalis* melanin composition revealed high concentrations of carbon, oxygen, sodium, and chlorine (Mbonyiryivuze et al., 2015). All but chlorine was observed in high concentrations in the melanin composition analysis from the strains herein studied. In fact, eumelanins have an abundance of carbon, oxygen but also nitrogen (Gonçalves et al., 2012). This element was not observed in our analysis. Only with this analysis, it would be expected that melanin from the Trichosporonaceae would be the DHN-type. To the best of our knowledge, the genome of *T. asahii* does not have a gene homologous to the polyketide synthase, the key gene for DHN-melanin production. In addition, the denaturant/hot acid treatment showed that the strains only produce melanin when L-DOPA is present. In fact, during the analysis of *S. officinalis* melanin using SEM-EDS, nitrogen was not detected in two of five replicates of the experiment, suggesting that the chemical composition is different depending on positions of the sample (Mbonyiryivuze et al., 2015).

Fungal melanins have the ability to bind to various metals, participating in metal bio absorption from rocks and other environmental niches (Cordero and Casadevall, 2017). Melanins have several potential sites to bind metal ions, and they are known for their high affinity to a variety of metals potentially toxic to fungi (García-Rivera and Casadevall, 2001). In this study, the melanin composition of two strains showed barium and vanadium, toxic elements present in the environment (Lazaridis et al., 2007; Menzie et al., 2008). Basidiomycetes are able to solubilize vanadium from mineral solids and immobilize dissolved vanadium through uptake and accumulation in mycelia or by the formation of extracellular crystals (Xu et al., 2019). This chemical element was found in the melanin composition of the *T. japonicum* strain. In addition, some basidiomycetes can accumulate high barium concentration (Demirbaş, 2001). As basidiomycetes produce different types of melanin (Toledo et al., 2017), our results suggest a possible role of this pigment in fungal tolerance to vanadium and barium.

Only the highly pathogenic species *T. asahii*, *T. inkin*, and *C. neoformans* presented iron in their melanin composition. During parasitism, iron availability is limited, and fungal pathogens may develop strategies to acquire this element (Saikia et al., 2014). The presence of iron in the major pathogenic Trichosporonaceae may indicate a role of melanin in iron homeostasis in these pathogens, conferring an advantage during infection over other less common trichosporonosis agents. These samples also presented copper and zinc in their composition. Both elements are important enzymatic cofactors needed during fungal parasitism (Culbertson et al., 2020; Alamir et al., 2021).

Characterization of the physicochemical properties of melanoid pigments has been carried out in different fungal species over the years. Magnetic and paramagnetic resonance studies, as well as the surface charge of this pigment, demonstrated the antioxidant capacity of melanin and its contribution with virulence (Polak, 1990; Casadevall et al., 2000; Sehnaz Alp, 2010; Cordero and Casadevall, 2017; Jeong-Joo et al., 2020). Our next steps will consist of determining how these biophysical properties favor the tissue damage cause by Trichosporonaceae species in mammalian host.

## Conclusion

At least seven human pathogenic species from the Trichosporonaceae family produces melanin when L-DOPA is available during growth. The composition and amount of melanin appear to vary within different strains. Biophysical properties of these melanins can confer to the Trichosporonaceae adaptive advantages to both parasitic and environmental conditions of fungal growth.

## Data Availability Statement

The original contributions presented in the study are included in the article/Supplementary Material, further inquiries can be directed to the corresponding author/s.

## Author Contributions

IA: research design, data analysis, and writing-original draft preparation and review and editing. GA: research design, data analysis, and writing-review and editing. FB-S and SF: study design, data analysis, and writing-review and editing. MF-C: research design and writing-review and editing. RZ-O: study design, data analysis, writing-review and editing, and funding acquisition. RA-P: study design, data analysis, and writing-original draft preparation and review and editing. All authors contributed to the article and approved the submitted version.

## Conflict of Interest

The authors declare that the research was conducted in the absence of any commercial or financial relationships that could be construed as a potential conflict of interest.

## Publisher’s Note

All claims expressed in this article are solely those of the authors and do not necessarily represent those of their affiliated organizations, or those of the publisher, the editors and the reviewers. Any product that may be evaluated in this article, or claim that may be made by its manufacturer, is not guaranteed or endorsed by the publisher.
