# Supplementary material for: Comparative Biophysical and Ultrastructural Analysis of Melanins Produced by Clinical Strains of Different Species From the Trichosporonaceae Family
Source: Front Microbiol. 2022 Apr 25;13:876611. doi: 10.3389/fmicb.2022.876611 (PMC9081797; doi:10.3389/fmicb.2022.876611)
Supplement: Supplementary file 1 [file Data_Sheet_1.docx]

Supplementary Material

## Supplementary Figures


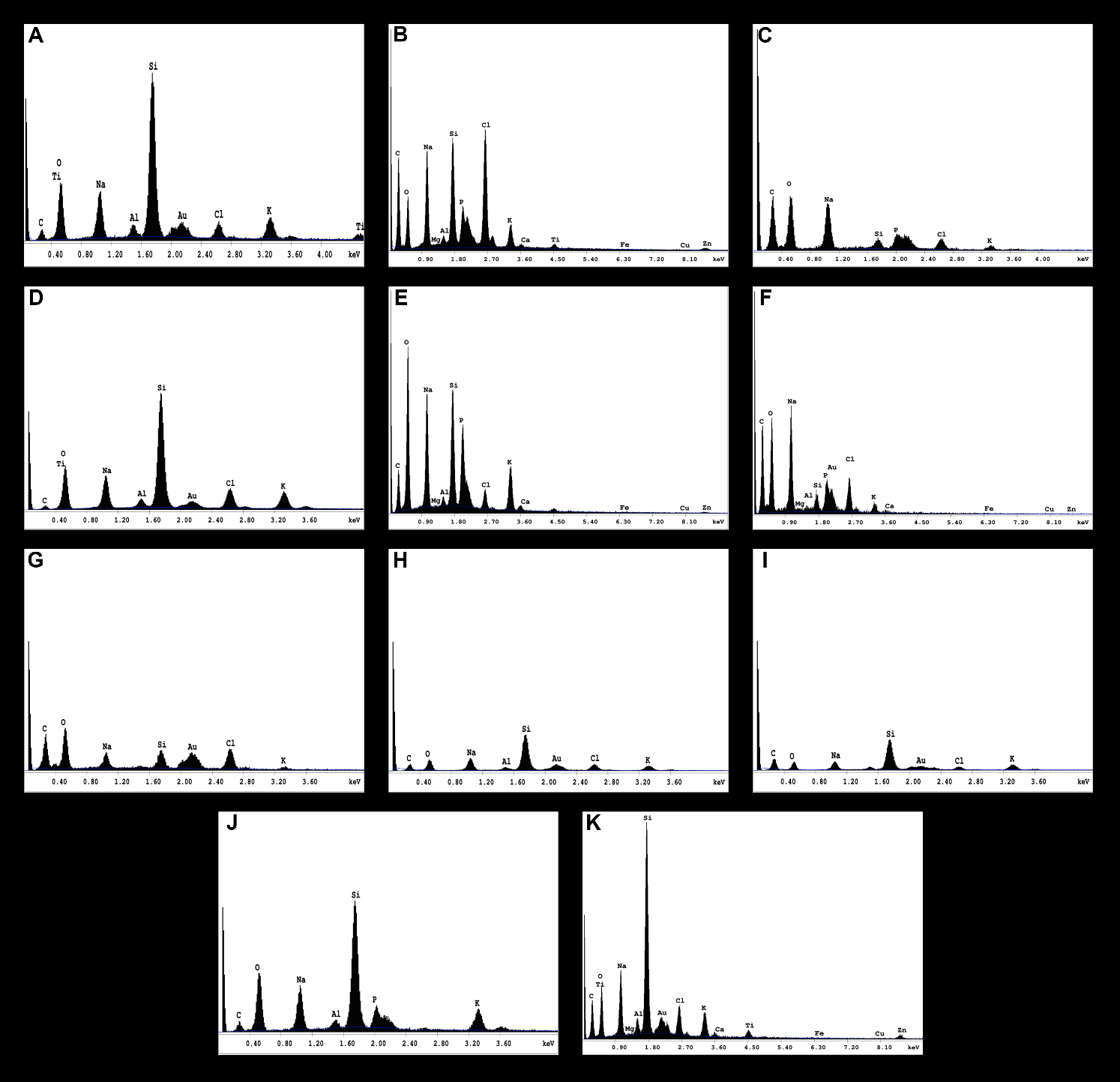


**Supplementary Figure 1.** **Energy dispersive spectroscopy (EDS) of melanins produced by clinical strains of different species from the Trichosporonaceae family.** **A)** *Trichosporon asahii* (CFP00944) **B)** *Trichosporon japonicum* (CFP00907) **C)** *Trichosporon faecale* (CFP00905) **D)** *Trichosporon inkin* (CFP00904) **E)** *Trichosporon inkin* (ATCC 18020) (CFP00946) **F)** *Trichosporon inkin* (CFP00951) **G)** *Apiotrichum montevideense* (CFP00950) **H)** *Apiotrichum montevideense* (CFP00909) **I)** *Cutaneotrichosporon debeurmannianum* (CFP00913) **J)** *Cutaneotrichosporon arboriformis* (CFP00914) **K)** *Cryptococcus neoformans* var. grubii H99

**
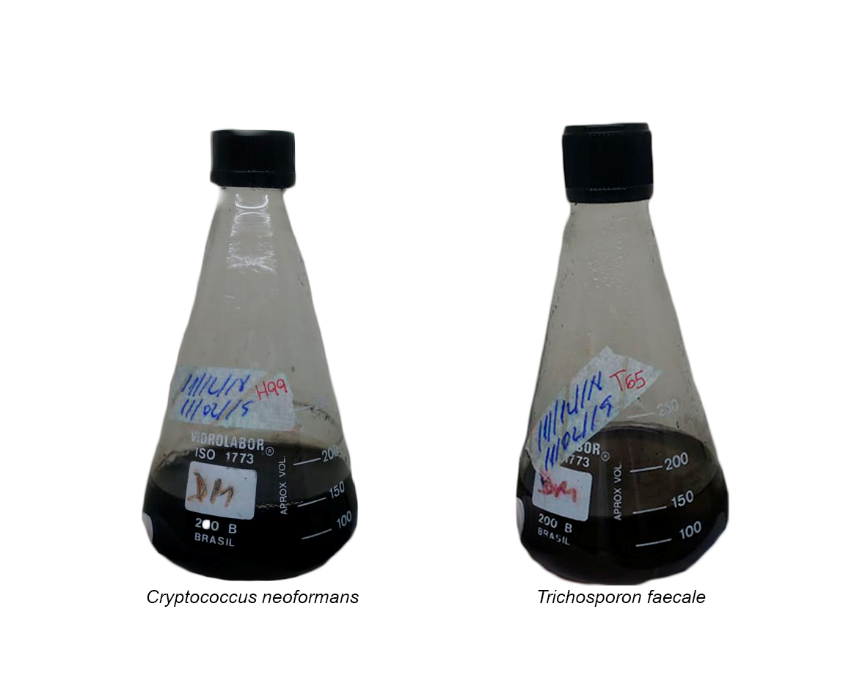
**

**Supplementary Figure 2.** Melanin production by cells of *Cryptococcus neoformans* and *Trichosporon faecale*
